# Oenological tannins mitigate rotenone-induced mitochondrial impairments and oxidative stress, with concomitant detection of urolithin A in the brain

**DOI:** 10.1016/j.bbrep.2025.102263

**Published:** 2025-09-12

**Authors:** Olga Wojciechowska, Michaël Jourdes, Mirosław Andrusiewicz, Małgorzata Pokrzywa, Marta Karaźniewicz-Łada, Jadwiga Jodynis-Liebert, Pierre-Louis Teissedre, Małgorzata Kujawska

**Affiliations:** a Department of Toxicology, Poznan University of Medical Sciences, Rokietnicka 3, 60-806, Poznan, Poland; b Unité de Recherche Œnologie, UMR 1366 INRAE, ISVV, Université de Bordeaux, 33882, Villenave-d'Ornon, France; c Department of Cell Biology, Poznan University of Medical Sciences, Rokietnicka 5D, 60-806, Poznan, Poland; d Airoptic Sp. z o.o., Rubiez 46B, 61-612, Poznan, Poland; e Department of Physical Pharmacy and Pharmacokinetics, Poznan University of Medical Sciences, Rokietnicka 3, 60-806, Poznan, Poland

**Keywords:** Ellagitannins, Mitochondria, Mitochondrial complex I, Mitochondrial membrane potential, Oxidative stress, Protein oxidation

## Abstract

Mitochondria are key organelles that supply energy to the brain, and their dysfunction contributes to neurotoxicity induced by environmental toxins such as rotenone. Recently, oenological tannins (OTs) and their colonic metabolite, urolithin A (UA), have been emphasized due to their potential neuroprotective activity. However, their role in counteracting toxin-induced mitochondrial impairments remains unclear. Therefore, this study aimed to investigate the administration of OTs to rotenone (ROT)-induced mitochondrial dysfunction and oxidative stress, key contributors to neurotoxicity. We measured mitochondrial membrane potential (MMP), the activity of mitochondrial complex I (Grishchuk et al.), and aldehyde dehydrogenase 2 (ALDH2) to assess mitochondria and protein carbonyl (PC) levels. We also checked the presence of UA in the brain. Our results indicate that the OTs treatment restored MMP, increased MCI and ALDH2 activity, and decreased PC content in ROT-induced rats. Furthermore, we confirmed the presence of UA in the brains of the animals. While its exact contribution to the observed mitochondrial effects remains undetermined, this finding suggests a potential role of the gut-derived metabolite in neuroprotection. Thus, we conclude that OTs administration attenuates mitochondria-related neurotoxicity. We call for further mechanistic studies and the putative contribution of metabolites, including UA, to the demonstrated mitoprotective effect of OTs treatment.

## Highlights

• Oenological tannins (OTs) mitigate rotenone-induced neurotoxicity.
• OTs restore mitochondrial function in a rat model.
• Urolithin A (UA) is detected in the brain after OTs treatment.
• OTs protect against oxidative damage in the brain.
• OTs may offer a potential therapeutic avenue.

## Graphical abstract

**Figure:**
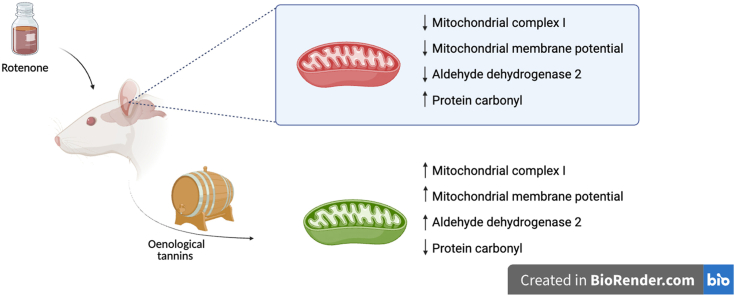

## 1 Introduction

The brain represents only 2 % of the body's weight but consumes 20 % of its total energy demand. Mitochondria are critical organelles for energy production via oxidative phosphorylation for cellular processes essential for neuronal growth, function, and regeneration [1]. Therefore, mitochondria directly affect the survival of cells, and maintaining their homeostasis ensures an adequate supply of energy to the brain. When their function is compromised, increased production of reactive oxygen species (ROS), oxidation and mutation of mtDNA, mitochondrial membrane potential (MMP) reduction, and mitochondrial permeability transition pore activation are observed. Moreover, mitochondrial dysfunctions contribute to the decreased activity of respiratory chain complexes, inhibited ATP production, and disturbances in mitophagy [2,3]. Mitophagy refers to the elimination of dysfunctional mitochondria in a selective autophagy process, thus increasing the quality of the cellular mitochondria pool [4]. Interference in the process leads to the accumulation of defective mitochondria, resulting in neuronal death and neurodegeneration [5]. One of the signals of impaired mitochondria, thus the precondition for mitophagy, is depolarization of the membrane below a certain MMP [6]. Chronic mitochondrial dysfunctions, combined with related bioenergetic failure, are critical contributors to neurodegenerative diseases [1].

Mitochondrial complex I [7] deficiency is implicated in various neurotoxic and neurodegenerative conditions, including but not limited to Parkinson's disease (PD) [8]. Rotenone (ROT) is a natural, plant-derived complex ketone used as a broad-spectrum insecticide, piscicide, and pesticide [9]. The compound can cross the blood-brain barrier and cellular membranes without any specific transporters because of the lipophilic nature of the toxin [10]. Mechanistically, ROT is also responsible for damaging the mitochondria, producing ROS, and inducing oxidative stress in the brain [11]. Exposure to the neurotoxin leads to loss of MMP, inhibition of MCI activity, leading to the depletion of ATP, and ultimately, nerve cell death [12]. Moreover, ROT exposure also causes increased lipid peroxidation and protein oxidative damage in targeted dopaminergic cells [13]. The role of oxidative stress in rotenone toxicity has been well established in the in vivo study [14]. While ROT is widely used as a neurotoxin model due to its ability to induce PD-like symptoms in rodents [15], its toxicity extends beyond the nervous system. Studies have demonstrated ROT-induced damage in multiple organs, including the liver, kidney, lung, spleen, stomach, and heart [16,17]. In this study, ROT was used to induce mitochondrial dysfunction and oxidative stress as a general neurotoxic model, rather than as a direct model of PD pathology. Given its continued use as a pesticide in certain regions, potential environmental exposure to ROT remains an important consideration [18].

As populations age and the burden of neurodegenerative diseases increases, the concept of brain health from the ageing and preventive care perspectives has emerged as the overarching strategy, especially in the context of a lack of established and validated disease-modifying options [19]. This idea is supported by increasing evidence for a link between lifestyle habits, such as diet and dietary components, and the occurrence of brain pathology. Focused attention has been given to the traditional Mediterranean diet, characterized by high consumption of plant foods and olive oil with low-to-moderate consumption of wine during meals [20]. For years, strong evidence has emerged of the positive impact of dietary polyphenols on health, especially against neurodegenerative diseases due to their numerous properties. Ellagitannins (ETs) are a diverse class of tannins found in several fruits and nuts. The polyphenols occur naturally in pomegranates, strawberries, raspberries, blackberries, walnuts, and wine [21]. Caruana and colleagues have reviewed experimental and clinical evidence on the putative role of wine polyphenols against brain pathology, highlighting their combined ability to suppress oxidative stress and neuroinflammation, modulate signalling pathways, decrease mitochondrial dysfunction, and affect amyloid aggregation [20]. Polyphenolic compounds, including ellagitannins, can be released from wood to the wine during maturation and ageing in oak barrels. Eight ellagitannins have been identified in traditional oak species: castalagin, vescalagin, granidin, and roburins (A, B, C, D, and E) [22]. There is also growing evidence of the contribution of secondary polyphenol metabolites, such as urolithins generated by colonic bacteria, to beneficial effects on human health, including neuroprotection [23]. To date, our knowledge about neuroprotective effects and biotransformation of the oenological tannins (OTs) is very scarce and limited to only a few studies [20,24]. Considering poor absorption of OTs along the gastrointestinal tract and their structural rearrangement by digestive enzymes and gut microbiota, it is suggested that smaller molecules of metabolites and catabolites like urolithins may be putative neurologically active derivatives contributing to the overall observed neuroprotective effect. It is known that ellagitannins, a class of hydrolyzable tannins, are hydrolyzed into ellagic acid (EA) during digestion due to the acidic conditions. EA can then be transformed by the gut microbiota to urolithins D, C, A, and B in the intestine and transported into blood circulation through intestinal epithelial cells as their lipophilicity increases compared to parent compounds [25]. Urolithin A (UA), the most abundant and widely studied colonic metabolite, is a beneficial compound providing anti-inflammatory, antiapoptotic, and antioxidant properties and neuroprotective effects in different health conditions [26,27]. Previously, we have demonstrated that UA is distributed to the brain [28]. In PD models, the administration of UA provided potentially favourable effects on brain health and symptom alleviation [[29], [30], [31]]. One of the key mechanisms of UA's action is involvement in mitochondrial function and mitophagy [32]. Further supporting this, recent research has provided additional evidence that urolithins can reach the brain. Ávila-Gálvez et al. [33] demonstrated that urolithins can be transported across the blood-brain barrier and may exert biological effects in neural tissues. However, the precise transport mechanisms and physiological relevance of these findings require further investigation. Urolithins, including UA, are considered active metabolites in individuals after intake of water-extracted standardized French *Quercus robur* wood extracts, commercially available as Robuvit®. Supplementation of Robuvit® is recommended for restoring higher levels of energy and activity to enhance physical endurance and performance [34].

Given the evidence provided above, our study aims to investigate whether OTs can mitigate rotenone-induced mitochondrial dysfunction and oxidative stress in the brain. Moreover, we examined whether OTs treatment was associated with detectable levels of UA in the brain, given its proposed role in mitochondrial health.

## 2 Materials and methods

### 2.1 Animals

The animal experiment was performed on six-week-old male Albino Wistar rats weighing 250–300 g and bred at the Toxicology Department of the Poznan University of Medical Sciences (Poznan, Poland). This strain and sex were selected based on our prior work demonstrating reproducible neurotoxic responses to rotenone [16], and due to their common use in neurotoxicological studies. Animals were conventionally kept (4 rats per cage) in polycarbonate cages (Tecniplast, Buguggiate, Italy) with wood shavings. The environmental temperature was 22 ± 2 °C with a relative humidity between 40 % and 54 % and 12/12-h light/dark cycle. Animals were fed *ad libitum* with commercial diets (labo feed H certified by ISO 22000) and drinking water.

### 2.2 Experimental design

The commercial OTs product − QUERTANIN® by Laffort (Bordeaux, France) was used in this experiment. The OTs product suspended in distilled water was given to rats intragastrically (*e.g.* at a dose of 100 mg/kg body weight (b.w.)/day). The tested dose was established based on preliminary experiments conducted to assess the tolerability and biological activity of the oenological tannins preparation used in this study. Beginning from the 11th day, rats were injected subcutaneously once daily for thirty-five days with ROT (Sigma-Aldrich, Poznań, Poland) in a dose of 1.3 mg/kg body weight, as previously validated in our toxicity model [16] to induce oxidative stress and related neurotoxicity. ROT was dissolved in helianthi oleum raffinatum (FAGRON a.s., Olomouc, Czech Republic), a vehicle previously used in our studies for its suitability for subcutaneous administration and ability to solubilize this lipophilic compound. Thirty-eight rats were randomly divided into four groups, as shown in Table 1.

**Table 1:** Rat groups and treatments. OTs, oenological tannins (QUERTANIN®), ROT, rotenone; *i.g*., intragastrically; *s.c.*, subqutaneous; b.w., body weight^1^(100 mg/kg b.w./day),^2^ (1.3 mg/kg b.w./day).

Group (n)	Treatment	
	1st–10th Day	11th–45th Day
Control (n = 8)	water (*i.g.)*	water (*i.g.)* + oleum (s.c.)
OTs^1^ (n = 11)	OTs^1^ (*i.g.)*	OTs^1^ (*i.g.)* + oleum (s.c.)
ROT^2^ (n = 8)	water (*i.g.)*	water (*i.g.)* + ROT^2^ (s.c.)
OTs + ROT (n = 11)	OTs^1^ (*i.g.)*	OTs^1^ (*i.g.)* + ROT^2^ (s.c.)

OTs, oenological tannins (QUERTANIN®), ROT, rotenone; *i.g*., intragastrically; *s.c.*, subcutaneous; b.w., body weight^1^ (100 mg/kg b.w./day), ^2^ (1.3 mg/kg b.w./day).

Twenty-four hours after the last treatment, the rats were euthanized with ketamine/xylazine (100 U/7.5 mg/kg b.w., intraperitoneally) and perfused intracardially with isotonic sodium chloride solution. Perfused brains were quickly removed, and the separated on-ice midbrains were snap-frozen using dry ice. For biochemical examinations, brain tissues were stored at −80 °C until further use. To determine UA in the brain, the whole brains of three rats from groups II and IV were collected after entire body perfusion with phosphate-buffered saline, pH 7.4, to avoid overlapping metabolites from the residual blood.

### 2.3 Determination of mitochondrial membrane potential (MMP)

Mitochondria were isolated from midbrain tissue under sterile conditions at 4 °C using the Mitochondria Isolation Kit for Tissue (PIERCE, Rockford, IL) according to the manufacturer's protocol. The MMP was measured using laser-scanning confocal microscopy after staining with tetramethylrhodamine methyl ester (TMRM, Cat. no. I34361, Thermo Fisher Scientific, Waltham, MA, USA) according to the manufacturer's instructions. Briefly, mitochondria adsorbed onto a glass-bottom dish were incubated with 50 nM TMRE in the dark for 10 min at room temperature. Fluorescence images were captured by a laser-scanning confocal microscope (Axio Observer. Z1 with laser scanning unit LSM780, Carl Zeiss GmbH, Germany), and the mean fluorescence intensity of TMRM was measured using ZEN2012 Blue Edition software (version 1.1.2.0, Carl Zeiss GmbH). Excitation wavelength 561 and emission filter 567–620 were applied. Relative MMP values were calculated by expressing fluorescence intensity as a percentage of the control group.

### 2.4 Biochemical examinations

Frozen midbrain tissues were homogenized using a handheld tissue homogenizer with a lysis buffer (Cell Lysis Buffer 2; Bio-Techne-R&D Systems, Minneapolis, MN, USA) and a cocktail of protease and phosphatase inhibitor (Protease Inhibitor Cocktail I, Bio-Techne-Tocris, Minneapolis, MN, USA) at a weight: volume ratio of 1:2. The homogenate was centrifuged at 10,000×*g* for 20 min at 4 °C. The supernatant was collected for the biochemical assays except for the mitochondrial aldehyde dehydrogenase 2 (ALDH2) activity assay.

Complex I activity was determined by following the oxidation of NADH to NAD+ and the simultaneous reduction of a dye detected spectrophotometrically at 450 nm using the Abcam Complex I Enzyme Activity Kit (ab109721, Abcam, Amsterdam, NL) per the manufacturer's directions.

The Protein Carbonyl (PC) content was quantified by the derivatization of protein carbonyl groups with 2,4-dinitrophenylhydrazine (DNPH), leading to the formation of stable dinitrophenyl (DNP) hydrazone adducts detected at 375 nm according to the manufacturer's protocol provided with the Protein Carbonyl Content Assay Kit (MAK094, Sigma-Aldrich, Poznań, Poland).

The activity of ALDH2 was determined using a colourimetric Mitochondrial Aldehyde Dehydrogenase (ALDH2) Activity Assay Kit (ab115348, Abcam, Amsterdam, NL). We followed the protocols provided by the manufacturer and used in our previous study [28].

The quantity of protein in samples was measured using the Bicinchoninic Acid Protein Assay Kit following the manufacturer's instruction (BCA1 AND B9643, Sigma-Aldrich, Poznań, Poland).

### 2.5 Dopamine (DA) level determination

As previously reported [35], we determined DA level by the homogenization of midbrain tissue with an extracting mixture (acetonitrile–0.1 M HCl–27 mM EDTA water solution), using a handheld tissue homogenizer and subsequently sonicated (20 min at 4 °C). Then, the sample was centrifuged at 6500×*g*, and the supernatant was filtered on a 0.2 mm PTFE microfilter before HPLC-MS analysis.

The analysis of DA employed a Shimadzu Nexera (Shimadzu Co., Kyoto, Japan) chromatograph and a triple quadrupole mass spectrometer, the LCMS-8030 (Shimadzu Co., Kyoto, Japan). We applied the Lab Solutions Series Workstation system (Shimadzu, Kyoto, Japan) for data processing.

DA was separated in a Gemini® C18 analytical column equipped with a security guard cartridge (Phenomenex, Torrance, CA, USA) and column oven (Shimadzu® Model CTO-2AC) to maintain the temperature of 25 °C. The mobile phase included an aqueous solution of acetic acid of pH = 2 (A) and a methanol (B) combination. We used the following gradient of elution: 0–3 min 5 % B, 3–5 min linear increase to 70 %, 5–8 min 70 % B, 8–10 min linear decrease to 5 %, 10–12 min 5 % B.

The mobile phase flow rate stood at 0.15 mL/min with 20 μL as the volume of the injected sample. The eluent from the liquid chromatography column was introduced to the MS detector using electrospray ionization in positive ion mode with the electrospray needle voltage of 4.5 kV. The temperature of the desolvation line, the heat block, and the interface were kept at 250 °C, 400 °C, and 350 °C, respectively. Nitrogen was employed as the nebulizing and drying gas with a flow rate of 2 and 10 L/min, respectively. The most sensitive mass transition was from *m*/*z* 154.1 to 136.9. Linearity of the method was confirmed for DA in the ranges of 0.5–10 ng/mL. The within-run and between-run precision was <13.7 % (expressed as relative standard deviations), and the within-run and between-run accuracy of the method was <14.5 % (expressed as the relative error) [35].

### 2.6 Urolithin A determination

UA was determined by the UPLC-ESI-QTOF-MS method following isolation from brain tissue according to a previously established protocol [28]. Briefly, the brains harvested from rats treated with QTs-treated group - groups II (n = 3) and IV (n = 3) were extracted with methanol: HCl (99.9:0.1 v/v) following enzymatic hydrolysis of conjugated UA metabolites according. The supernatant was evaporated, re-suspended in methanol, and filtered through a 0.45 μm PVDF filter (Merc, Warszawa, Poland) before analysis by UPLC-ESI-QTOF-MS. The concentration of UA was quantified based on the calibration curve performed for UA (SML1791, Sigma-Aldrich, Poznan, Poland).

### 2.7 Statistical analysis

The experiments were performed in duplicate, and 8 animals per experimental group were used. For the analysis of urolithin A distribution, 3 animals were used. Data are presented as mean ± SD or median ± IQR. For parametric variables, the differences between the groups were analyzed by one-way analysis of variance (ANOVA), followed by the uncorrected Fisher's LSD post hoc test. The groups' differences were analyzed with Kruskal–Wallis for non-parametric variables, followed by Dunn's post hoc test. Differences were considered significant at p < 0.05. All statistical analyses and charts were performed using PRISM 10 software (GraphPad Software Inc., La Jolla, CA, USA).

## 3 Results

### 3.1 OTs treatment improved mitochondrial function

**Fig. 1:**
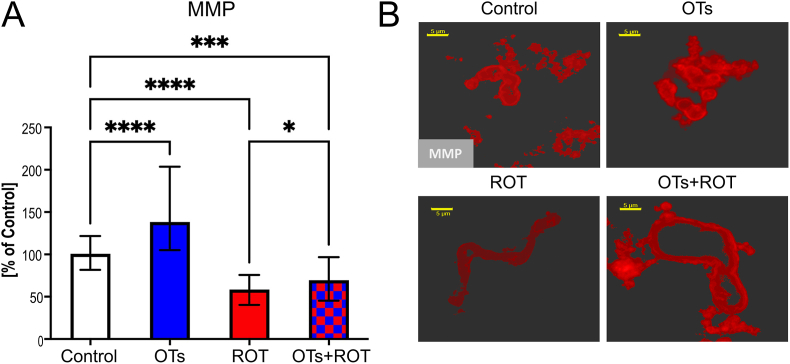
Effect of oenological tannins (OTs) treatment on Mitochondrial Membrane Potential (MMP) in the midbrain of rotenone (ROT)-injected rats **(A)** Graph shows the relative mean fluorescence intensity of tetramethylrhodamine methyl ester (TMRM), expressed as a percentage of the control group. Data are presented as median ± IQR (n = 70 images/group) and analyzed using Kruskal–Wallis followed by Dunn's post hoc test. ∗∗∗∗p < 0.0001; ∗∗∗p < 0.001; ∗p < 0.05. **(B)** Representative fluorescence images of mitochondrial membrane potential (TMRM fluorescence intensity). Higher fluorescence intensity corresponds to greater integrity. The scale bar represents 5 μm. Rotenone (ROT) significantly reduces MMP compared to the control, while OTs treatment significantly increases MMP compared to the ROT group and even higher than the control group. Brighter fluorescence (higher MMP) in the control and OTs-treated groups, diminished fluorescence in the ROT-injected group, and restored fluorescence in the OTs + ROT-treated group.

To examine the role of OTs treatment in protecting against mitochondrial dysfunctions, we determined MMP and the activity of MCI in the midbrain. As shown by the fluorescence intensity of tetramethylrhodamine methyl ester (TMRM), which is a cell-permeant dye sequestered by active mitochondria with intact membrane potentials, animals injected with ROT exhibited a significant decline, by 41 %, in the level of MMP compared to the control. Treatment with OTs reversed this ROT-induced mitochondrial impairment/defect by 17 %. Interestingly, OTs administered alone caused an increase in MMP by 52 % (Fig. 1). ROT significantly reduces MMP compared to the control, while OTs treatment significantly increases MMP compared to the ROT group, and even higher than the control group. TMRM fluorescence intensity was directly proportional to MMP. The images visually confirm the quantitative data in, showing brighter fluorescence (higher MMP) in the control and OTs-treated groups, diminished fluorescence in the ROT-injected group, and restored fluorescence in the OTs + ROT-treated group. While the exact mechanism remains unclear, some polyphenols have been reported to influence mitochondrial function, including membrane potential stabilization [36,37]. Further studies are needed to determine whether this effect results from direct mitochondrial interactions or secondary metabolic adaptations.

**Fig. 2:**
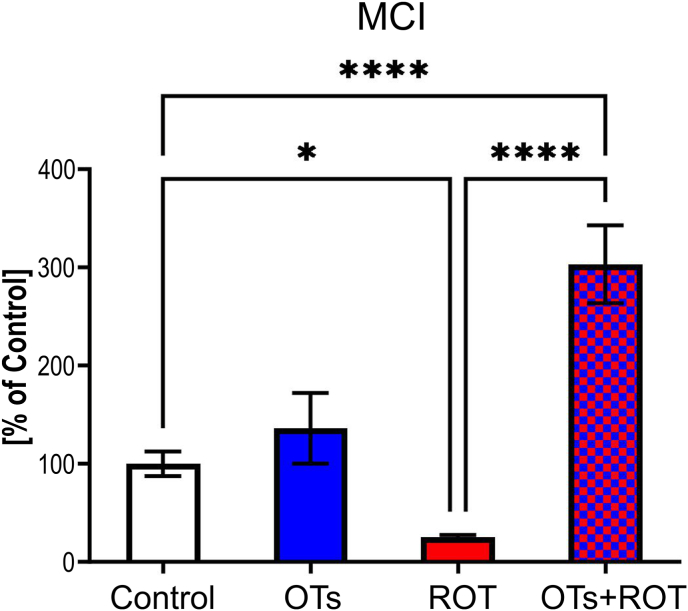
Effect of oenological tannins (OTs) treatment on mitochondrial complex I (MCI) activity in the midbrain of rotenone (ROT)-injected rats. Data are presented as mean values ± SEM of eight rats per group and analyzed using one-way analysis of variance (ANOVA) followed by Fisher's LSD test. ∗∗∗∗p < 0.0001; ∗p < 0.05. Rotenone significantly decreases MCI activity, while OTs treatment significantly increases MCI activity in ROT-injected rats, suggesting a protective effect. OTs alone slightly increase the MCI activity.

Given that ROT is an inhibitor of MCI, we investigated whether ROT-induced inhibition of this enzyme could be prevented by OTs treatment. We found that ROT inhibited MCI, which was markedly, almost threefold elevated upon OTs treatment (Fig. 2). In this study, we measured MCI activity as a functional indicator of complex I, which includes NADH dehydrogenase as a key component. This assay provides an integrated measure of NADH oxidation and electron transfer efficiency, reflecting the overall function of the enzyme complex rather than isolated protein levels. The increase in MCI activity following OTs treatment was observed alongside mitochondrial dysfunction induced by ROT. While this suggests a potential adaptive response, further studies are needed to determine its functional significance.

### 3.2 OTs treatment protected against ROT-induced decrease in mitochondrial ALDH2 activity and protein oxidative damage

**Fig. 3:**
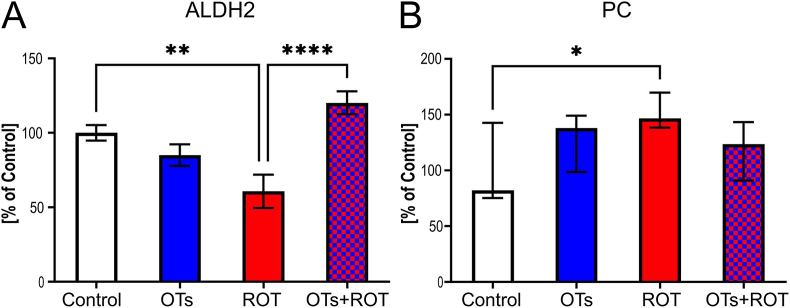
Effect of oenological tannins (OTs) treatment in the midbrain of rotenone (ROT)-injected rats on **(A)** mitochondrial aldehyde dehydrogenase 2 (ALDH2) activity. Data are presented as mean values ± SEM of eight rats per group and analyzed using one-way analysis of variance (ANOVA) followed by Fisher's LSD test. **(B)** protein carbonyl (PC) content. Data are presented as median ± IQR and analyzed using Kruskal-Wallis followed by Dunn's multiple comparisons test. ∗∗∗∗p < 0.0001 ∗∗p < 0. 01; ∗p < 0.05. ALDH2 activity is significantly reduced in the ROT-injected group, while OTs treatment significantly restores ALDH2 activity in ROT-injected rats. While PC content increases in the ROT-injected group, OTs treatment reduces PC content in the ROT-injected rats.

Aldehyde dehydrogenase 2 (ALDH2) was analyzed as a key mitochondrial enzyme involved in the detoxification of reactive aldehydes and the regulation of redox homeostasis. ALDH2 activity is critical for maintaining mitochondrial function, as its impairment has been linked to increased oxidative stress and metabolic dysfunction [38,39]. Given the role of ALDH2 in mitochondrial health, we assessed whether OTs treatment could influence its activity in rotenone-exposed animals. As shown in Fig. 3, OTs administration to ROT-injected rats caused an increase of 49 % in ALDH2 activity in the midbrain, which was similar to the control level. This effect was accompanied by the normalization of elevated, by ROT injection, protein oxidative damage attributed to protein carbonyl content. While OTs treatment reduced PC levels relative to ROT, this decrease was not statistically significant. However, PC levels in the OTs-treated group did not exceed those observed in the control group, suggesting a normalization effect. To further evaluate oxidative stress, we also measured glutathione (GSH) levels. However, no significant differences were observed between the experimental groups (see Supplementary Fig. S1).

### 3.3 OTs treatment effect on dopamine (DA) level

ROT primarily induces neurotoxicity by damaging dopaminergic neurons, leading to disruptions in the dopaminergic system. Given this, dopamine (DA) levels were measured as an indirect marker of dopaminergic neuron damage to assess the potential neuroprotective effects of OTs treatment. Since DA depletion reflects neuronal loss or dysfunction in dopaminergic pathways, its evaluation provides insight into the extent of ROT-induced neurotoxicity and the potential protective mechanisms of OTs.

**Fig. 4:**
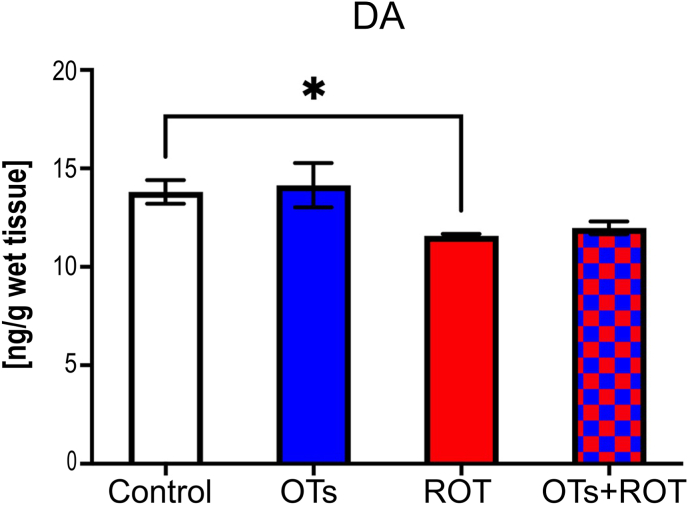
Effect of oenological tannins (OTs) on dopamine (DA) levels in the midbrains of rotenone (ROT)-injected rats. Data are presented as mean values ± SEM of eight rats per group and analyzed using one-way analysis of variance (ANOVA) followed by Fisher's LSD test. ∗p < 0.05 vs. control. Dopamine levels in the ROT-injected group are significantly decreased compared to the control. There is no significant increase in dopamine after the administration of OTs in the ROT-injected rats. OTs alone slightly increase the DA levels.

As shown in Fig. 4, ROT treatment led to a significant reduction in DA level compared to the control. While OTs treatment alone had no significant effect, the OTs + ROT group showed no significant difference from either the control or ROT groups. This pattern suggests a potential trend toward normalization of dopamine levels by OTs, possibly attenuating the ROT-induced deficit without exceeding baseline levels.

### 3.4 UA presence in the brains of OTs-treated rats

The presence of UA in brain tissue was confirmed by UPLC-UV-QTOF-MS analysis (Fig. S3). The concentrations of UA in the brains of rats administered with OTs alone and in combination with ROT were 0.69 ± 0.20 ng/g wet tissue and 2.74 ± 3.0 ng/g wet tissue, respectively, calculated as a mean value ± SD of three rats from the relevant groups. These findings confirm that UA, a gut-derived metabolite of ellagitannins, can cross the blood-brain barrier. However, further studies are needed to determine whether UA directly contributes to the observed mitoprotective effects.

## 4 Discussion

Our study examines the impact of OTs administration on mitochondrial impairment in ROT-intoxicated rats. ROT, as a commonly used neurotoxin, is responsible for mitochondrial dysfunction. The compound is a well-established inhibitor of MCI of the mitochondrial respiratory chain, disturbing the electron transfer from the iron-sulfur centres to ubiquinone, thus disturbing the oxidative phosphorylation process and resulting in decreased ATP production [40,41]. Moreover, incomplete electron transition increases ROS formation. Diminished respiratory activity, combined with possible impairment of mitochondrial DNA, lipid peroxidation, and protein oxidative damage, leads to apoptotic cell death [42,43]. The neurotoxic effects of the rotenone regimen used here have been extensively validated in our previous studies employing the same dose and schedule [16,28], which consistently demonstrated mitochondrial dysfunction, oxidative stress, and dopaminergic impairment in the midbrain. In the present study, we observed a similar profile, confirming the reproducibility of this neurotoxic model and providing a robust basis for evaluating the protective effects of OTs. On the other hand, the biological significance of polyphenols is primarily due to their antioxidative properties related to scavenging free radicals and ROS and enhancing endogenous cytoprotective mechanisms, thus preventing the cells from oxidation [44]. They inhibit neural degeneration caused by rotenone by augmenting the antioxidant defence system and reducing mitochondrial dysfunction, as outlined in the literature review [45,46].

The consumption of ETs, due to their antioxidative, anti-inflammatory, and mitoprotective properties, has been investigated in various conditions, including neurodegenerative, cardiovascular diseases, cancer, and other oxidative stress-related chronic disorders [21,47,48]. Pomegranate is a rich source of ETs, specifically punicalagin and EA, that are considered to contribute to the overall health benefits reported for the fruit [49]. Administration of pomegranate peel extract microparticles activated mitochondrial complex IV and preserved mitochondrial cristae structure in brown adipose tissue in mice fed on a high-fat diet (HFD), suggesting elevated oxidative phosphorylation system activity [48]. Cao et al. [47] indicated that punicalagin could enhance mitochondrial oxidative capacity, which was attributed to increased activity of mitochondrial complex I and complex II in the heart tissue of HFD rats treated with pomegranate extract. While the precise mechanism remains unclear, polyphenols have been reported to influence mitochondrial function, including the activity of respiratory chain complexes. Previous studies indicate that polyphenols can mitigate the decline in mitochondrial complex I activity induced by oxidative stress [50]. Further research is needed to determine whether this effect results from direct interactions with the electron transport chain or secondary metabolic adaptations. Moreover, pomegranate supplementation normalized mitochondrial metabolism and prevented the initiation of apoptosis, accompanied by the activation of the AMP-activated protein kinase pathway [47]. Similar outcomes were obtained from punicalagin-enriched pomegranate extract on HFD-induced nonalcoholic fatty liver disease in rats, contributing to elevated activity of mitochondrial complex I, II, and IV and replenished content of ATP in the liver [51]. In rats with hyperlipidemia-induced metabolic changes, punicalagin increased the protein levels of mitochondrial complexes I, II, and IV, alleviating mitochondrial dysfunction of the thoracic aorta [52]. In HepG2 cells, exposure to punicalagin alleviated loss of MMP, ROS production, and ATP depletion caused by palmitate-induced lipotoxicity [53].

The class of ETs, as hydrolyzable phenolic compounds, can be hydrolyzed under physiological conditions during digestion into EA. Studies have investigated the multiple therapeutic properties of this compound, including pleiotropic and cell-type-specific effects on mitochondrial functions [54]. Treatment with EA alleviated apoptosis associated with mitochondrial dysfunction in arsenic-induced neuroinflammation in rats. EA restored MMP in a dose-dependent manner after a decrease linked to arsenic exposure [55]. Pre-incubation with EA of rat embryonic fibroblasts exposed to H_2_O_2_ increased the activity of mitochondrial complexes I, II, and IV, confirming its protective and antioxidative role [50], while in isolated rat heart mitochondria with bevacizumab-induced cardiotoxicity, it elevated only the activity of mitochondrial complex II [56]. In ROT-induced mitochondria-related neurotoxicity in *Drosophila melanogaster*, EA supplementation protected against MCI inhibition and alteration in the bioenergetics caused by the neurotoxin. Significantly, the mitoprotective effect of EA rescued the ROT-induced neurotoxic phenotype in the flies [57].

In the central nervous system, the respiration and redox balance of the mitochondria are associated with their ability to detoxify lipid aldehydes such as 4-hydroxynonenal (HNE), malondialdehyde (MDA), and neurotransmitter-derived aldehydes such as dihydroxyphenylacetaldehyde (DOPAL). Leiphon et al. [58] demonstrated that mitochondrial ALDH2 is a sensitive target of pesticide inactivation, with rotenone decreasing the detoxification of HNE. This is consistent with our previous findings showing decreased mitochondrial ALDH2 activity in the brains of ROT-intoxicated rats [16,28]. Pharmacological activation of mitochondrial ALDH2 prevented ROT-induced MMP depolarization, ROS overproduction, and subsequent activation of the mitochondrial apoptotic pathway in SH-SY5Y cells and primary cultured SN dopaminergic neurons [59]. ALDH2 plays an important role in maintaining mitochondrial homeostasis, and its inhibition seems to be one of the mechanisms through which environmental toxicants promote neurodegeneration [60]. In our previous study, pomegranate juice administration preserved midbrain ALDH2 activity, providing substantial protection against oxidative damage, suppression of lipid peroxidation, and enhancement of neuronal survival with subsequent neuroprotective effects in the rotenone rat model of PD [28,35].

The current study provides valuable insights into the protective effects of oenological tannins against ROT-induced mitochondrial dysfunction. Our results demonstrated that OTs treatment significantly attenuated the ROT-induced decline in mitochondrial membrane potential (MMP) and restored the activity of mitochondrial complex I [7] and aldehyde dehydrogenase 2 (ALDH2). The restoration of MMP by OTs suggests a stabilization of the inner mitochondrial membrane, which is crucial for maintaining the proton gradient necessary for ATP synthesis. Rotenone, an MCI inhibitor, disrupts the electron transport chain, leading to a collapse of MMP and reduced ATP production. The fact that OTs treatment reversed this effect indicates that OTs may either directly interact with the electron transport chain to improve its efficiency or indirectly protect mitochondria from ROT-induced damage. Our study also revealed that OTs treatment increased ALDH2 activity, which is critical for the detoxification of reactive aldehydes, including those produced during lipid peroxidation. ALDH2 plays a vital role in maintaining redox homeostasis within mitochondria, and its impairment has been associated with increased oxidative stress and metabolic dysfunction. The restoration of ALDH2 activity by OTs suggests that OTs may help mitigate oxidative damage within mitochondria, thereby supporting their function. This is consistent with the observed reduction in PC levels, although the decrease was not statistically significant compared to the ROT group alone. A more pronounced reduction in PC levels with OTs treatment would provide stronger evidence of its antioxidant effects.

Furthermore, the detection of urolithin A in the brain suggests a potential role for UA in mediating the observed mitochondrial effects. While the exact contribution of UA remains undetermined, its presence in the brain implies that it may cross the blood-brain barrier and directly influence mitochondrial function. UA has been shown to enhance mitophagy, the selective removal of damaged mitochondria, thereby promoting mitochondrial turnover and improving cellular health.

It is clear that the mitochondrial impairment observed preferentially in midbrain neurons, especially in the substantia nigra pars compacta region, in ROT-treated rats corresponds to oxidative stress and related oxidative damage to cellular macromolecules [61]. A disturbed oxidative state triggers glycoxidation reactions, including modifications of free amino groups in proteins with carbonyl groups of reducing sugars. This results in the overproduction of advanced glycation end products (AGEs), a heterogeneous group of molecules [62,63]. The process disturbs the structure and function of proteins and enhances oxidative stress [63,64]. Glycation of mitochondrial proteins in *Caenorhabditis elegans* was responsible for the organelle's functional impairment, ROS production, and the induction of apoptotic pathways [65]. Overproduction of PC groups is one of the biomarkers of oxidative stress associated with aging and various diseases [66]. In the in vitro model, pomegranate extract, its single phenolic constituents (EA, punicalagin, gallic acid) decreased the formation of AGEs by scavenging reactive carbonyl species (RCS) [67]. RCS are chemicals with highly reactive carbonyl groups, which can modify proteins, nucleic acids, and aminophospholipids, resulting in cytotoxicity and mutagenicity [68]. In the in vitro study by Viljanen et al. [69], raspberry extract, also containing ETs, showed strong antioxidant activity toward protein oxidation, attributed to the inhibition of protein carbonyl compound formation and lipid oxidation in liposomes. PC and MDA levels in the rotenone-challenged flies were declined by exposure to EA [70]. In mice with MPTP-induced mitochondrial defects, EA injection ameliorated the oxidative stress, as decreased MDA levels in the striatum tissue were noticed, thus preventing dopamine neuron degeneration [70]. EA treatment also reduced the 6-hydroxydopamine-induced lipid peroxidation [[71], [72], [73]], and ROS production in the brain of experimental animals [71].

Consistent with this, a decreased content of protein carbonyls accompanied by elevated activity of mitochondrial ALDH2 in the midbrain of ROT-injected rats upon treatment with OTs was observed in this study. Similar effects we reported in ROT-injected rats co-treated with rich in condensed tannins − cranberry juice [16,74].

In addition to its effects on mitochondrial function, OTs treatment, which resulted in UA detection in the brain, showed improved olfactory discrimination learning in the ODT (see Supplementary Fig. S2). While not conclusive, this suggests a potential functional benefit, warranting further investigation in dedicated behavioral studies. Since ROT primarily induces neurotoxicity by damaging dopaminergic neurons [75], DA levels were measured as an indirect marker of dopaminergic neuron integrity. ROT treatment led to a significant reduction in DA levels compared to the control. OTs treatment alone had no significant effect, whereas the OTs + ROT group showed no significant difference from either the control or ROT groups. This pattern suggests a potential trend toward normalization of DA level by OTs, possibly attenuating the ROT-induced deficit without reaching baseline levels. The lack of significant changes in DA levels suggests that OTs may not have fully counteracted ROT-induced neuronal damage. Further studies are needed to determine the extent of their neuroprotective potential and whether additional mechanisms contribute to their observed effects.

Multiple therapeutic properties of ETs could be linked to their colonic conversion to urolithins by several bacterial genera. UA, the most abundant and widely studied ETs’ colonic metabolite, has been investigated due to its involvement in mitochondrial homeostasis [27]. The neuroprotective effects of treatment with pomegranate juice were observed in our previous study and were probably linked to UA, which was detected in the brains of the animals [28]. Exposition of human chondrocytes from healthy and osteoarthritis donors to UA improved mitochondrial respiration [76]. Lipopolysaccharide-induced BV2 microglial cells showed improvement in mitochondrial functions and attenuation of the proinflammatory response upon UA exposition. The treatment caused an increase in MMP [30]. On the other hand, Esselun et al. [77] observed no effect of UA on autophagy in SH-SY5Y-APP695, with limited impact on mitochondrial function, suggesting its hormetic effects on several genes related to mitochondrial biogenesis at the transcriptional level. Moreover, UA has a beneficial role against oxidative stress, enhancing the endogenous antioxidant response and inhibiting ROS overproduction [27,78] with observed decreased formation of AGEs by scavenging RCS [67].

An extensive review of clinical effects and the possible mechanism of action of using French oak wood extract Robuvit has been performed. *Quercus robur* wood extract is a potent source of polyphenols belonging to the ETs class, including castalagin, vescalagin, grandinin, roburin E, ellagic acid, and gallic acid. Supplementation with Robuvit was linked to increased levels of urolithins A, B, and C and the number of their colonic producers. Improved mitochondrial function and a decreased level of oxidative stress in blood plasma were observed upon Robuvit supplementation [34]. A gene expression analysis after Robuvit intake in healthy subjects revealed the elevation in mitochondrial protein NADH-dehydrogenase, the enzyme involved in the electron transport chain [79]. The results of this work will shed light on the understanding of the role of OTs in neuroprotection due to their mitoprotective and antioxidative properties. The detection of UA in the brain suggests that gut-derived metabolites of OTs may contribute to the observed mitochondrial effects. However, since our study did not directly assess UA's functional role, further research is required to establish its precise contribution. Interestingly, the higher levels of UA observed in the brains of ROT + OTs-treated rats compared to OTs alone may be partly attributed to the decreased brain mass in animals exposed to ROT, which could lead to relatively higher UA concentrations when expressed per gram of tissue. This observation underscores the need for cautious interpretation of tissue-normalized metabolite levels. Therefore, extended research on the UA treatment alone against rotenone-induced mitochondrial impairments is required. Moreover, the metabolic fate of OTs and their implications for mitochondrial functions should be considered in further studies.

While we did not directly measure oxidative stress in this study, we assessed several key indicators related to oxidative stress and mitochondrial function, including protein carbonyl levels. We quantified protein carbonyl content, a marker of protein oxidation, and observed that OTs treatment reduced PC content in ROT-induced rats. This suggests a reduction in oxidative damage to proteins. We also measured MMP, a key indicator of mitochondrial health and function. Rotenone is known to induce oxidative stress, leading to a decline in MMP. OTs treatment restored MMP, indicating a protective effect against rotenone-induced mitochondrial dysfunction. Additionally, ALDH2 is a key mitochondrial enzyme involved in the detoxification of reactive aldehydes, which are products of oxidative stress. OTs treatment increased ALDH2 activity, suggesting an enhanced capacity to detoxify reactive aldehydes. Although we found UA detection, we did not directly assess UA's functional role. Our findings suggest that the Gut-derived metabolite can contribute to observing mitoprotective effects. These findings, while indirect, suggest that OT treatment mitigates oxidative stress. We agree that direct measurement of oxidative stress markers would further strengthen our conclusions. However, we believe that the current data provide compelling evidence for the protective effects of OTs against rotenone-induced mitochondrial impairments and related oxidative damage.

Although we observed that OTs treatment influenced mitochondrial function and oxidative stress parameters, further research is warranted to deepen our understanding of the underlying mechanisms. The detection of UA in the brain opens new avenues to explore its potential modulatory role in mitochondrial regulation, particularly in the context of rotenone-induced neurotoxicity. Future studies may benefit from isolating and analyzing UA's effects to better delineate its neuroprotective relevance.

## 5 Conclusions

Our findings suggest that OTs administration alleviated the mitochondria-related neurotoxicity caused by ROT. OTs treatment restored MMP and activity of MCI and ALDH2 while diminishing PCs caused by the neurotoxin. Thus, we conclude that OTs administration attenuates mitochondria-related neurotoxicity. Moreover, we found that this effect is accompanied by colonic metabolite UA in the brain. This study demonstrated its occurrence in the midbrain of OTs-administered rats, so further experimental investigations on the putative contribution of UA to the observed mitochondrial-related improvement should be undertaken.

## CRediT authorship contribution statement

**Olga Wojciechowska:** Writing – review & editing, Writing – original draft, Formal analysis. **Michaël Jourdes:** Validation, Software, Methodology, Investigation, Data curation. **Mirosław Andrusiewicz:** Validation, Software, Methodology, Investigation, Data curation. **Małgorzata Pokrzywa:** Validation, Software, Methodology, Investigation, Data curation. **Marta Karaźniewicz-Łada:** Methodology, Investigation. **Jadwiga Jodynis-Liebert:** Supervision, Conceptualization. **Pierre-Louis Teissedre:** Supervision, Methodology, Conceptualization. **Małgorzata Kujawska:** Writing – review & editing, Writing – original draft, Validation, Supervision, Software, Project administration, Methodology, Investigation, Funding acquisition, Formal analysis, Data curation, Conceptualization.

## ARRIVE statement

The study was carried out in compliance with the ARRIVE guidelines.

## Compliance with ethical standards

The study protocol was approved by the Local Ethics Committee on the Use of Laboratory Animals in Poznan, Poland (14/2018, Apr 27, 2018).

## Funding

The research was funded by 10.13039/501100004281Narodowe Centrum Nauki, grant numbers: 2017/26/D/NZ7/00748 and 2024/53/N/NZ7/02236.

## Declaration of competing interest

The authors declare that they have no known competing financial interests or personal relationships that could have appeared to influence the work reported in this paper.

## Data Availability

Data will be made available on request.
