# Supplementary material for: Oenological tannins mitigate rotenone-induced mitochondrial impairments and oxidative stress, with concomitant detection of urolithin A in the brain
Source: Biochem Biophys Rep. 2025 Sep 12;44:102263. doi: 10.1016/j.bbrep.2025.102263 (PMC12803790; doi:10.1016/j.bbrep.2025.102263)
Supplement: Multimedia component 1 [file mmc1.docx]

# **Materials and Methods**

## Determination of reduced glutathione (GSH) level

The reduced glutathione (GSH) level was quantified with Ellman’s reagent and measured spectrophotometrically as previously described (Kujawska et al., 2019).

## Olfactory Discrimination Task (ODT)

As we characterized in our previous study (Kujawska et al., 2021), the test was held in a box (60 cm × 40 cm × 50 cm) with two interconnected compartments enabling free movement. One compartment included sawdust with an odor familiar to the animal, while the second one was filled with clean sawdust, designated as a non-familiar odor. After being placed in the middle of the box, the animal was free to explore each part, while being recorded for 5 minutes. An exploration of both sections equally indicates olfactory impairment, while the preference to investigate a particular compartment suggests intact olfactory function.

The discrimination index (DI) was calculated by dividing the difference in exploration time between the two compartments (non-familiar − familiar) by the total exploration time for both compartments (non-familiar + familiar), expressed as a percentage. Positive DI scores indicate a preference towards non-familiar, while negative – familiar odors (Kujawska et al., 2021).

# **Results**

## OTs treatment effect on GSH level

To further evaluate oxidative stress in our study, we also measured glutathione (GSH) levels. However, no significant differences were observed between the experimental groups (Figure S1).

**
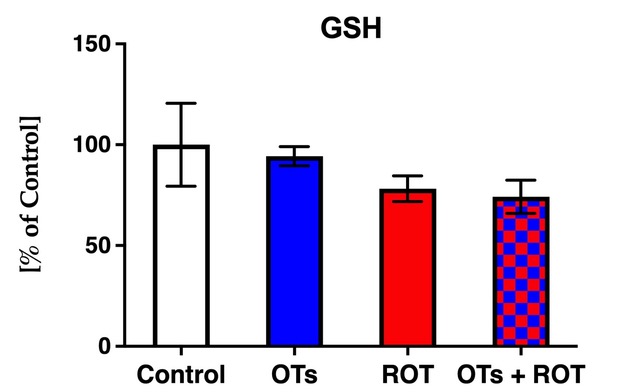
**

**Figure S1.** Effect of oenological tannins (OTs) treatment on reduced glutathione (GSH) level in the midbrain of rotenone (ROT)-injected rats. Data are presented as mean values ± SEM of eight rats per group and analyzed using one-way analysis of variance (ANOVA) followed by Fisher’s LSD test.

## OTs treatment effect on the Olfactory Discrimination Task (ODT)

While the primary focus of the study was on mitochondrial dysfunction at the cellular and biochemical levels, we also acknowledge the importance of functional outcomes. As part of our exploratory behavioral assessment, we performed the Olfactory Discrimination Task (ODT) to evaluate sensory processing, which can be affected by rotenone-induced neurotoxicity (Figure S2). OTs treatment significantly improved olfactory discrimination learning exhibited to increased DI index.


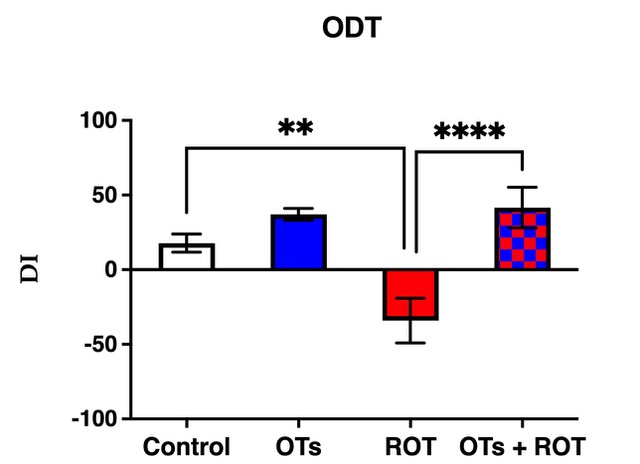


**Figure S2.** Effect of oenological tannins (OTs) treatment on olfactory discrimination task (ODT) expressed as olfactory discrimination index (DI) in rotenone (ROT)-injected rats. Data are presented as mean values ± SEM of eight rats per group and analyzed using one-way analysis of variance (ANOVA) followed by Fisher’s LSD test. ** p < 0.01 vs. Control. **** p < 0.0001 vs. ROT.

## 2.3. Urolithin A determination

***
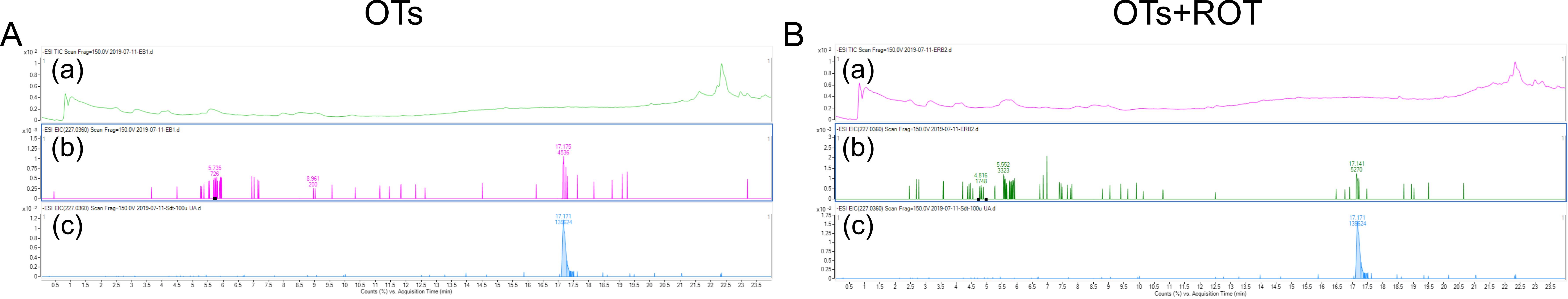
***

**Figure S3.** Representative raw UPLC-UV-QTOF chromatograms for a brain sample from rats treated with **(A)** oenological tannins (OTs) alone and (**B**) OTs combined with rotenone (ROT), showing: (a) total ion chromatogram, (b) selected ion chromatogram of *m*/*z* 227.0360 corresponding to urolithin A (UA), and (c) selected ion chromatogram of *m*/*z* 227.0360 for pure UA standard**.**
